# Extracellular Matrix Metalloproteinase Inducer (EMMPRIN) promotes lung fibroblast proliferation, survival and differentiation to myofibroblasts

**DOI:** 10.1186/s12931-016-0334-7

**Published:** 2016-02-17

**Authors:** Nadia A. Hasaneen, Jian Cao, Ashleigh Pulkoski-Gross, Stanley Zucker, Hussein D. Foda

**Affiliations:** Department of Medicine and Research, Veterans Administration Medical Center, Northport, USA; Department of Medicine, Stony Brook Medicine, Stony Brook, New York USA; Division of Pulmonary, Critical Care and Sleep Medicine, Stony Brook University Medical Center, Stony Brook, NY 11794-8172 USA

**Keywords:** EMMPRIN, TGF- β1: lung fibroblast, Apoptosis, WNT/ β-catenin

## Abstract

**Background:**

Idiopathic pulmonary fibrosis (IPF) is a chronic progressively fatal disease. Extracellular Matrix Metalloproteinase Inducer (EMMPRIN) is a glycosylated transmembrane protein that induces the expression of some matrix metalloproteinase (MMP) in neighboring stromal cells through direct epithelial–stromal interactions. EMMPRIN is highly expressed in type II alveolar epithelial cells at the edges of the fibrotic areas in IPF lung sections. However, the exact role of EMMPRIN in IPF is unknown.

**Methods:**

To determine if EMMPRIN contributes to lung fibroblast proliferation, resistance to apoptosis, and differentiation to myofibroblasts, normal Human lung fibroblasts (NHLF) transiently transfected with either EMMPRIN/GFP or GFP were treated with TGF- β1 from 0 to 10 ng/ml for 48 h and examined for cell proliferation (thymidine incorporation), apoptosis (FACS analysis and Cell Death Detection ELISA assay), cell migration (Modified Boyden chamber) and differentiation to myofibroblasts using Western blot for α–smooth actin of cell lysates. The effect of EMMPRIN inhibition on NHLF proliferation, apoptosis, migration and differentiation to myofibroblasts after TGF- β1 treatment was examined using EMMPRIN blocking antibody. We examined the mechanism by which EMMPRIN induces its effects on fibroblasts by studying the β-catenin/canonical Wnt signaling pathway using Wnt luciferase reporter assays and Western blot for total and phosphorylated β-catenin.

**Results:**

Human lung fibroblasts overexpressing EMMPRIN had a significant increase in cell proliferation and migration compared to control fibroblasts. Furthermore, EMMPRIN promoted lung fibroblasts resistance to apoptosis. Lung fibroblasts overexpressing EMMPRIN showed a significantly increased expression of α- smooth muscle actin, a marker of differentiation to myofibroblasts compared to control cells. TGF-β1 increased the expression of EMMPRIN in lung fibroblasts in a dose-dependent manner. Attenuation of EMMPRIN expression with the use of an EMMPRIN blocking antibody markedly inhibited TGF-β1 induced proliferation, migration, and differentiation of fibroblasts to myofibroblasts. EMMPRIN overexpression in lung fibroblasts was found to induce an increase in TOPFLASH luciferase reporter activity when compared with control fibroblasts.

**Conclusion:**

These findings indicate that TGF-β1 induces the release of EMMPRIN that activates β-catenin/canonical Wnt signaling pathway. EMMPRIN overexpression induces an anti-apoptotic and pro-fibrotic phenotype in lung fibroblasts that may contribute to the persistent fibro-proliferative state seen in IPF.

## Background

Idiopathic pulmonary fibrosis (IPF) is a progressive interstitial lung disease with median survival time of about 3 years from initial diagnosis [1]. IPF is characterized by the progressive and ultimately excessive accumulation of mesenchymal cells (including fibroblasts and their differentiated phenotype, myofibroblasts) and extracellular matrix in the lung [2]. An important feature of IPF is the presence of fibroblastic foci throughout the entire lung [3]. Several studies have demonstrated a paucity of apoptosis of the fibroblasts within the fibroblastic foci in IPF [4, 5]. Moreover, fibroblasts from fibrotic lungs are resistant to a variety of apoptotic stimuli [6, 7]. IPF fibroblasts also acquire an invasive phenotype and are able to invade artificial basement membranes more readily than normal fibroblasts [8]. TGF-β1 is strongly implicated in the pathogenesis of lung fibrosis. TGF-β1 has been shown to promote the induction of an apoptosis resistant phenotype of fibroblasts [9, 10]. Furthermore, it is a critical regulator of fibroblast differentiation into activated myofibroblast [11, 12]. TGF- β1 is secreted by several cells including type II alveolar epithelial cells (AEC), and usually secreted in an inactive form attached to a latency-associated peptide [13]. Certain MMPs such as MMP-2, MMP-9, and MT1-MMP have been shown to activate latent TGF- β1 [14–18].

Extracellular matrix metalloproteinase inducer (EMMPRIN), also known as CD147 or basigin is a trans-membrane glycoprotein expressed on epithelial cells that is responsible for the induction of MMPs in neighboring stromal cells through direct epithelial–stromal interactions [19–22]. EMMPRIN is expressed in type II alveolar epithelial cells at the edges of the fibrotic areas in IPF lung sections [23] and its expression is associated with an induction of MMP-1, -2, -3, -9 as well as α-SMA in the neighboring stromal area [24] implying a role of EMMPRIN in IPF epithelial-stromal interactions [22, 23, 25]. Recently, more direct evidence demonstrated the involvement of EMMPRIN in fibroblast differentiation to myofibroblasts by inducing α-SMA expression in an MMP independent manner [22, 25]. Furthermore, EMMPRIN has been shown to have a regulatory role over the Wnt/β -catenin signaling pathway [26]. Silencing EMMPRIN inhibited β -catenin signaling, cell migration, proliferation, anchorage-independent growth and tumor growth in a mouse tumor xenograft model [26]. WNT/ β -catenin signaling pathway has been shown to play a major role in the development of experimental and human pulmonary fibrosis [27].

In this study we demonstrate that EMMPRIN overexpression in human lung fibroblasts contributes to a pro-fibrotic phenotype of fibroblasts by inducing fibroblast proliferation, survival, migration and differentiation to myofibroblast possibly through activating β-catenin/canonical Wnt signaling pathway.

## Methods

### Cell culture

Normal human lung fibroblasts (NHLF) were purchased from Lonza Walkersville Inc. (Walkersville, MD) and were cultured in Dulbecco’s modified Eagle medium (DMEM) (Invitrogen, Carlsbad, CA, USA) with 10 % fetal bovine serum, and 1 % penicillin/streptomycin (Invitrogen). Cells from passages 5 to10 were used in all experimental studies.

### Transfection of EMMPRIN

Transient transfection of NHLF with EMMPRIN was achieved as previously described [28].

- *Construction of the plasmids.*

A 1.6 kb length of cDNA representing the entire EMMPRIN sequence encoding 269 amino acid residues was placed in an *Eco*RI site under the control of the cytomegalovirus (CMV) promoter in pcDNA3 (a mammalian expression vector used for efficient expression; Invitrogen). To facilitate identification of transfected cells in vitro, green fluorescent protein (GFP) GFPmut1variant cDNA (Clontech Laboratory, Inc., Palo Alto, CA, USA) was inserted into the EMMPRIN-containing plasmid. The GFP cDNA along with a separate upstream CMV promoter from enhanced GFP plasmid-C1 were inserted into EMMPRIN expression vectors. An additional polyadenylation signal from pSG5 (Stratagene, La Jolla, CA, USA) was placed downstream of the EMMPRIN gene to provide balanced expression of both recombinant genes under control of CMV promoters. The resulting plasmids were named EMMPRIN/GFP. As a control plasmid, GFP cDNA alone was subcloned into pcDNA3 without EMMPRIN cDNA.

b) *Transient transfection of human lung fibroblasts.*

Human lung fibroblasts grown in 35 mm tissue-culture plates were cultured in 2 ml 10 % serum-DMEM media and incubated for 24 h. with 150nM NaOH containing polyethylenimine (PEI), and pcDNA, expressing EMMPRIN complexes, or control vector. The cells were then washed with PBS and cultured in DMEM media containing 10 % FBS. The efficiency of transfection was based on immunoblotting assay using EMMPRIN monoclonal antibodies (Chemicon International, Temecula, CA) and MMP release using gelatin zymography to assess MMP-2 and -9.

### TGF-β1 and EMMPRIN functional blocking antibody treatment

NHLF were plated in 6 well plates in DMEM supplemented with 10 % FCS and cultured until 70 – 80 % confluent. Cells were subjected to starvation by washing cells twice with serum free DMEM for 2 h, followed by the addition of DMEM and 0.01 % bovine serum albumin (BSA) to each well. Cells were treated with TGF- β1 (R&D, Minneapolis, MN) in a dose from 0 to 10 ng/ml for 24 h. In some experiments, NHLF were induced to undergo programmed cell death by incubation with 50nM staurosporine (STS) for 3 h prior to the end of the experiment. Additionally, NHLF were cultured in the presence of either the EMMPRIN functional blocking antibody (100 ng/ml) or IgG control antibody (Research Diagnostics, Inc., Flanders, NJ) to confirm the effects of EMMPRIN on NHLF cells.

### Apoptosis assays

- *Annexin V-FITC/propidium iodine (PI) staining:* NHLF were treated for 24 h in serum-free medium under the described conditions. Detached cells were collected by centrifugation. Cells were resuspended in Annexin V binding buffer (BD Biosciences, San Diego, CA, USA). Cells were then incubated with Annexin V-FITC and PI for 15 min at room temperature. Cells were analyzed and quantified by flow cytometry.
- *Cell Death Detection ELISA assay:* Fragmentation of DNA after cell death was determined by photometric enzyme immunoassay (Cell Death Detection ELISA^PLUS^, Roche Applied Science) per manufacturers instruction. Briefly, NHLF from the different treatment conditions were lysed using lysis buffer and centrifuged at 200 × *g*, and cytoplasmic fractions were transferred to streptavidin-coated plates that had been incubated with a biotinylated, monoclonal anti-histone antibody. The amount of fragmented DNA of nucleosomes bound to anti-histone antibody was evaluated by peroxidase-conjugated monoclonal anti-DNA antibody and the plates were read at 405 nm on a spectrophotometer.

### Proliferation assays

NHLF from different treatment conditions were examined for cell proliferation using [^3^H] Thymidine incorporation to assess DNA synthesis rates. Eight hours prior to the end of the experiment, 1 μCi/ml [3H] thymidine was added to NHLF. After 8 h of incubation at 37 °C, the conditioned media were aspirated. The cells were washed twice with PBS at 4 °C, and cold 5 % trichloroacetic acid was added for 30 min to precipitate protein and DNA. The precipitates were washed with cold water and re-suspended in 0.5 ml 1 M NaOH, and then 0.4 ml aliquots were added to 4 ml scintillation fluid and counted in a scintillation counter (Packard Instrument, Downers Grove, IL, USA).

### Cell viability assays

NHLF from each experimental group were examined for cell viability using Cell Titer-Glo® Luminescent Cell Viability assay (Promega Corporation, Madison, WI) following the manufacturer’s instructions.

### Migration assay using a modified Boyden chamber assay

NHLF overexpressing EMMPRIN or GFP were examined for their ability to migrate in the presence TGF-β1 using a modified Boyden chamber assay. The migration assays were performed with transwell (Costar, Corning, NY, USA) 24-well tissue-culture plates composed of polycarbon membranes with 8 μm pores. EMMPRIN or GFP lung fibroblasts were seeded on the upper chambers of the transwells at 1 × 10^5^ cells in 100 μl DMEM media containing 0.1 % BSA. TGF-β1 in doses of 0 to10 ng/ml was added to the lower chambers. The transwells were incubated for 24 h at 37 °C in a CO_2_ incubator. The number of cells that migrated to the lower surface of the membrane was counted under 200× magnification. Ten high-power, random fields were counted per sample. Each group was run in triplicate.

### Western blotting

Protein concentration of cell lysates was determined by BCA. Equal amounts of proteins (20 μg) of cell lysates were resolved in 8-16 % SDS-PAGE and transferred onto a nitrocellulose membrane (Amersham Biosciences, Pittsburgh, PA). After blocking with 5 % milk, the membranes were incubated with primary antibodies overnight at 4 °C followed by incubation with horseradish peroxidase-conjugated secondary antibodies and detection by use of an enhanced chemiluminescence detection system (Amersham Biosciences, Pittsburgh, PA). Primary antibodies included mouse monoclonal anti- α-SMA (1:10000 dilution, Sigma Aldrich), anti-human EMMPRIN (1:1000 dilution, Santa Cruz Biotechnology), rabbit polyclonal anti-Caspase 3 (1:500 dilution, Santa Cruz Biotechnology), rabbit polyclonal anti β-Catenin (1:1000 dilution, Abcam, Cambridge, MA) and β-Tubulin (1:200 dilution; Santa Cruz Biotechnology). Band densities were digitalized and quantified using image analysis software. Results were expressed as a ratio of band density to total β-tubulin.

### Gelatin zymography

Gelatin zymography of conditioned media was performed as described previously [28]. Briefly, conditioned media from the different treatment conditions were diluted 1:1 in non-reducing sample buffer and separated on 10 % SDS polyacrylamide gels containing 0.1 % gelatin (Invitrogen) for 150 min at 125 V. SDS was removed by incubation with renaturing buffer (Triton X-100, 2.5 % diluted in water) for 30 min at room temperature. The gels were washed for 30 min in developing buffer (Invitrogen) and then incubated overnight at 37 °C in fresh developing buffer. Finally, gels were stained with Coomassie blue. Zones of enzymatic (gelatinolytic) activity were characterized by the absence of Coomassie blue.

### Wnt luciferase reporter assays

NHLF overexpressing EMMPRIN/GFP or GFP alone were plated at a density of 5x10^4^ cells/well in a 12-well plate. Cells were transfected with TOPFLASH or FOPFLASH and a renilla luciferase plasmid (Promega, Madison, WI) as a control for transfection efficiency. Transfection of plasmid DNA into cells was achieved using polyethylenimine (PEI) and NaCl (Biosciences, San Jose, CA). Cells were cultured in DMEM containing 10 % FBS. TGF-β1 was added to the fibroblasts one day after transfection. Cells were harvested 72 h after transfection, and luciferase activity was assayed using the Dual Luciferase Assay kit (Promega, Madison, WI) according to the user’s manual. Luciferase activity was normalized to renilla activity.

### Statistical analysis

All results were reported as mean ± SEM. ANOVA for repeated measures was used to assess differences amongst conditions when multiple time-points were compared. Students’ t- test for unpaired data was used to assess the difference between conditions. P < 0.05 was considered to be significant.

## Results

### EMMPRIN overexpression in human lung fibroblasts induces a pro-fibrotic phenotype of fibroblasts

**Fig. 1:**
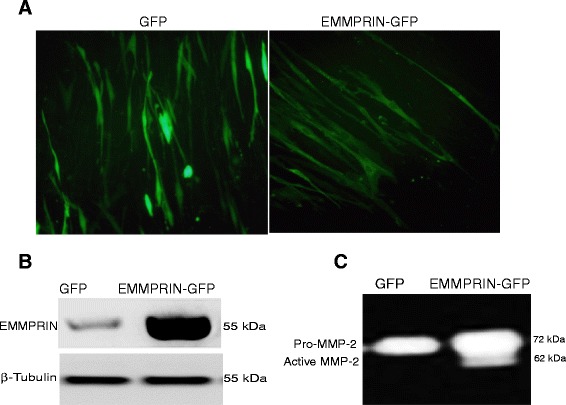
EMMPRIN overexpression in normal human lung fibroblasts (NHLF): NHLF were transiently transfected with either EMMPRIN/GFP or GFP alone and assessed for EMMPRIN expression using (**a**) Fluorescent microscopy demonstrating the distribution of GFP throughout the cell (left panel) in GFP control cells, while EMMPRIN-GFP cells primarily display cell-surface GFP expression (right panel). White Bar = 50 um. **b** Western blot analysis and (**c**) Gelatin zymography for MMP-2 release and activation (n = 10)

**Fig. 2:**
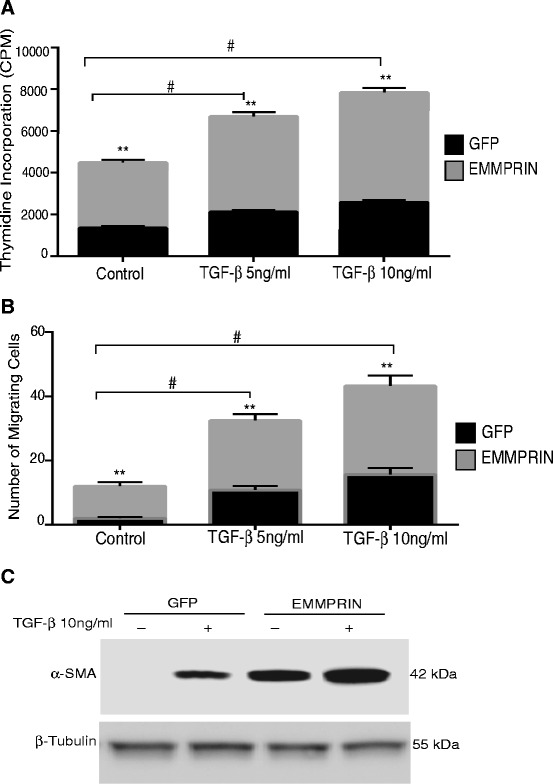
EMMPRIN overexpression induces phenotypic changes in normal human lung fibroblasts. Growth arrested NHLF transiently transfected with either EMMPRIN/GFP or GFP were treated with TGF- β1 5 and 10 ng/ml for 48 h. **a** Cell proliferation was assessed by thymidine incorporation. **b** Cell migration using Modified Boyden chamber. Each bar represents means ± SEM of 3 independent experiments each run in duplicates. ** p < 0.001 comparing GFP to EMMPRIN/GFP transfected cells, # p < 0.01 Comparing TGF- β1 treated cells to control. **c** A representative Western blot for α – smooth actin (α-SMA) of cell lysates from EMMPRIN/GFP and GFP control cells in the presence and absence of TGF-β1. β-Tubulin was used as the loading control. Image is representative of 3 independent experiments each run in duplicate

EMMPRIN overexpression was confirmed in NHLF cells by detecting GFP using fluorescent microscopy, protein expression of EMMPRIN via western blot, and MMP-2 activation using gelatin zymography (Fig. 1a-c). As expected, NHLF cells overexpressing EMMPRIN exhibited a significant increase in cell proliferation as evidenced by increased thymidine incorporation at 48 h compared to control GFP cells (p < 0.001; Fig. 2a). TGF-β1, in a dose-dependent manner, significantly increased thymidine incorporation into both EMMPRIN overexpressing fibroblasts and GFP control cells (p < 0.001; Fig. 2a).

To examine the effects of EMMPRIN on the migratory capacity of the lung fibroblasts, the cell migration assay was carried out using a modified Boyden chambers with 8-μm pore membrane inserts. EMMPRIN overexpression in NHLF induced a significant increase in cell migration when compared to control GFP stable cells (p < 0.001; Fig. 2b). TGF-β1, in a dose-dependent manner, significantly increased cell migration of both EMMPRIN overexpressing fibroblasts and GFP control cells (p < 0.05; Fig. 2b).

Differentiation of lung fibroblasts to myofibroblasts was assessed by analyzing α- smooth actin. Our results demonstrate that NHLF overexpressing EMMPRIN had a significant increase in α- smooth actin expression compared to GFP control cells. Further, TGF-β1 significantly increased expression of α- smooth actin in EMMPRIN overexpressing NHLF compared to GFP transfected control cells (Fig. 2c).

### EMMPRIN induces resistance of human lung fibroblasts to apoptosis

**Fig. 3:**
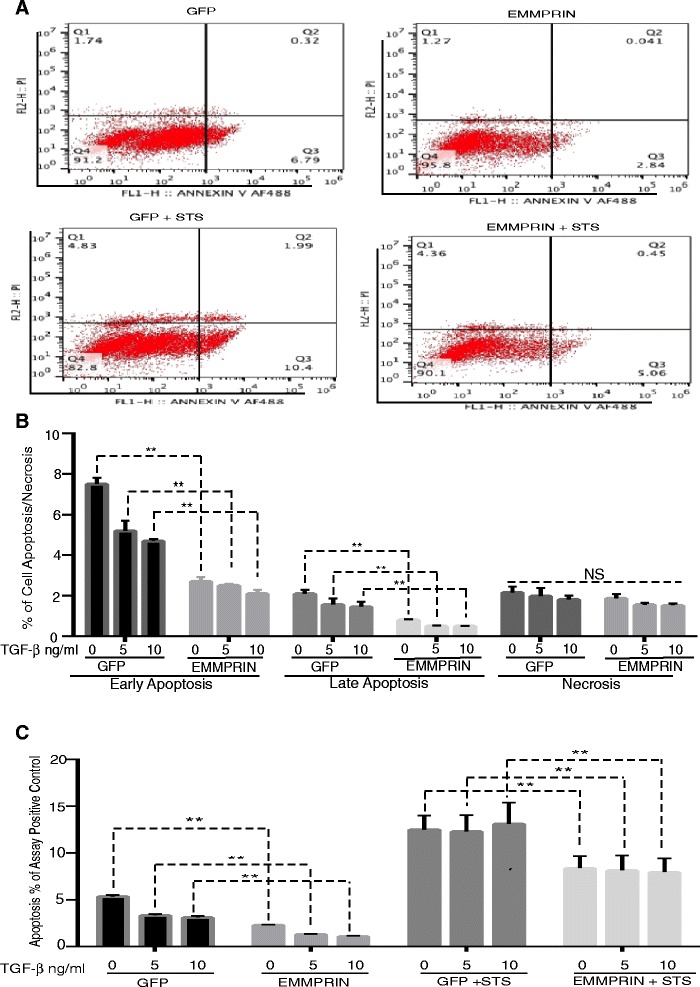
EMMPRIN overexpression induces resistance of normal human lung fibroblasts to apoptosis: Growth arrested NHLF overexpressing either EMMPRIN/GFP or GFP were treated with TGF- β1 from 0 to 10 ng/ml for 24 h in the presence and absence of 0.5 mM of Staurosporine (STS) added for 3 h prior to the end of the experiments. Apoptosis was measured by FACS analysis (**a**-**b**) using annexin V/PI staining and cell death detection ELISA assay (**c**). Relative apoptosis is expressed as a percentage of the assay-positive control that was run on the ELISA plate for each experiment. All samples were run in triplicate for each ELISA and FACS analysis. ** p < 0.001 comparing GFP to EMMPRIN/GFP transfected cells (n = 3 independent experiments)

To determine the effect of EMMPRIN on NHLF survival, EMMPRIN/GFP and GFP transfected NHLF treated with TGF- β1 were examined for apoptosis using FACS analysis and a cell death detection ELISA assay. As shown in Fig. 3a-c, EMMPRIN overexpression in NHLF inhibited both (Annexin V^+^/PI^-^) early and (Annexin V^+^/PI^+^) late apoptosis as compared to GFP control NHLF. There was no significant difference in (Annexin V^-^/PI ^+^) cell necrosis between EMMPRIN overexpressing NHLF and GFP control cells (Fig. 3a & b). TGF- β1 significantly inhibited both early and late apoptosis only in GFP transfected NHLF (p < 0.05; Fig. 3b & c). Next we examined the effect of EMMPRIN on fibroblast survival during STS induction of apoptosis. STS is a broad spectrum protein kinase inhibitor that inhibits numerous Ser/Thr and Tyr kinases, which triggers cell death. STS has been shown to induce fibroblast apoptosis in cells from patients with pulmonary fibrosis [29, 30]. Treatment of NHLF overexpressing EMMPRIN with STS resulted in a significant decrease in the percentage of apoptotic cells compared to GFP control cells treated with STS (Fig. 3a and c). TGF-β1 treatment had no significant effect on the percentage of apoptosis of both GFP and EMMPRIN transfected NHLF in the presence of STS (Fig. 3c). This finding shows that EMMPRIN inhibits fibroblast apoptosis in the presence of apoptotic stimulus independent of TGF- β1.

### TGF–β1 increases EMMPRIN expression and MMP-2 activation in human lung fibroblasts

**Fig. 4:**
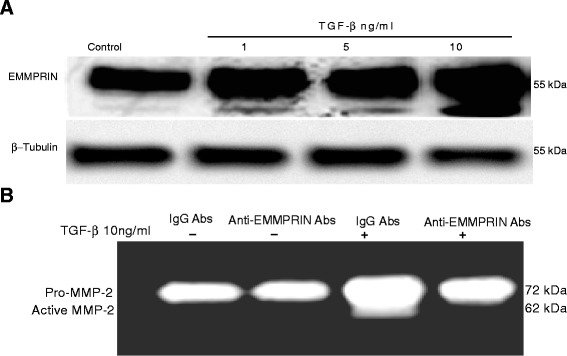
TGF-β1 induces EMMPRIN Expression and MMP-2 activation in normal human lung fibroblasts; inhibition of MMP-2 activation by EMMPRIN functional blocking antibody: (**a**) A representative Western blot showing the expression of EMMPRIN in the cell lysates from NHLF stimulated with TGF-β1 compared to control untreated cells. β-Tubulin was used as the loading control. **b** A representative zymography demonstrating MMP-2 activation in the conditioned media from NHLF stimulated with TGF-β1 compared to the control untreated cells and inhibition of MMP-2 activation by EMMPRIN functional blocking antibody (n = 3 independent experiments, each run in duplicate)

We examined in these experiments whether TGF-β1 induced NHLF proliferation, migration and differentiation was associated with EMMPRIN expression. Our results demonstrated that exposing NHLF to TGF-β1 was associated with an increase in the expression of EMMPRIN (Fig. 4a), MMP-2 activation (Fig. 4b).

### EMMPRIN functional blocking antibody induces apoptosis in human lung fibroblasts

**Fig. 5:**
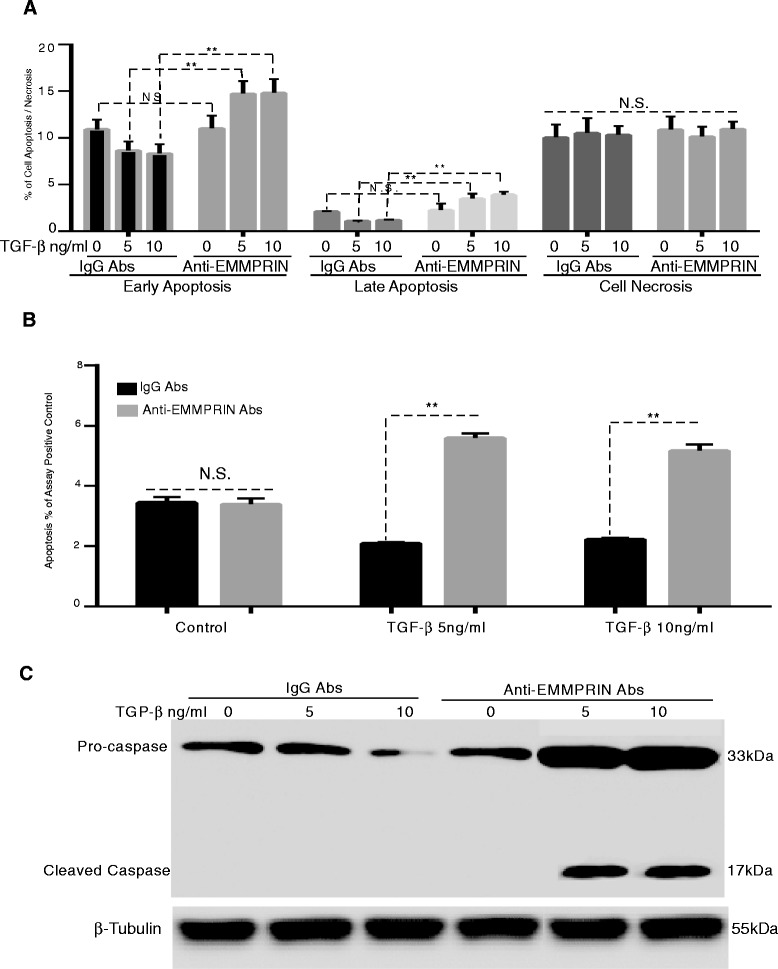
EMMPRIN functional blocking antibody induces apoptosis in normal human lung fibroblasts: NHLF treated for 24 h with TGF- β1 in the presence of either EMMPRIN functional blocking antibody or IgG control antibody. Apoptosis was measured by (**a**) FACS analysis using annexin V/PI staining; (**b**) Cell Death Detection ELISA assay, each bar represents means ± SEM of 3 independent experiments, each run in duplicate, ** p < 0.001 comparing EMMPRIN functional blocking antibody to IgG control antibody treated cells; and (**c**) Western blot for caspase- 3 of cell lysates from NHLF treated with TGF-β1 (5 and 10 ng/ml) in the presence of EMMPRIN functional blocking antibody or IgG control antibody. β-Tubulin was used as the loading control. Image is a representative of 3 independent experiments, each run in duplicate

Finding that TGF-β1 induces EMMPRIN expression in NHLF, we sought to determine if EMMPRIN inhibition could enhance NHLF susceptibility to apoptosis. NHLF were treated with TGF- β1 in the presence of either EMMPRIN functional blocking antibody or IgG antibody for 24 h. TGF-β1 inhibited apoptosis of NHLF in a dose dependent manner. EMMPRIN functional blocking antibody also abrogated the effect of TGF-β1 on NHLF. The EMMPRIN functional blocking antibody induced an increase in the percentage of early and late apoptotic NHLF only after TGF- β1 treatment (Fig. 5a and b). EMMPRIN blocking antibody treatment of NHLF abrogated the survival effect of TGF- β1 on NHLF and restored the expression of caspase 3 protein close to control levels (Fig. 5c). To confirm that the EMMPRIN functional blocking antibody inhibits EMMPRIN induction by TGF- β1, we examined MMP-2 release and activation in the conditioned media using gelatin zymography. Our results showed that TGF- β1 treatment induced an increase in MMP-2 release/activation and EMMPRIN functional blocking antibody inhibited MMP-2 activities returned it to control levels (Fig. 4b).

### TGF-β1 induces human lung fibroblast proliferation, migration and differentiation to myofibroblast: Inhibition by EMMPRIN functional blocking antibody

**Fig. 6:**
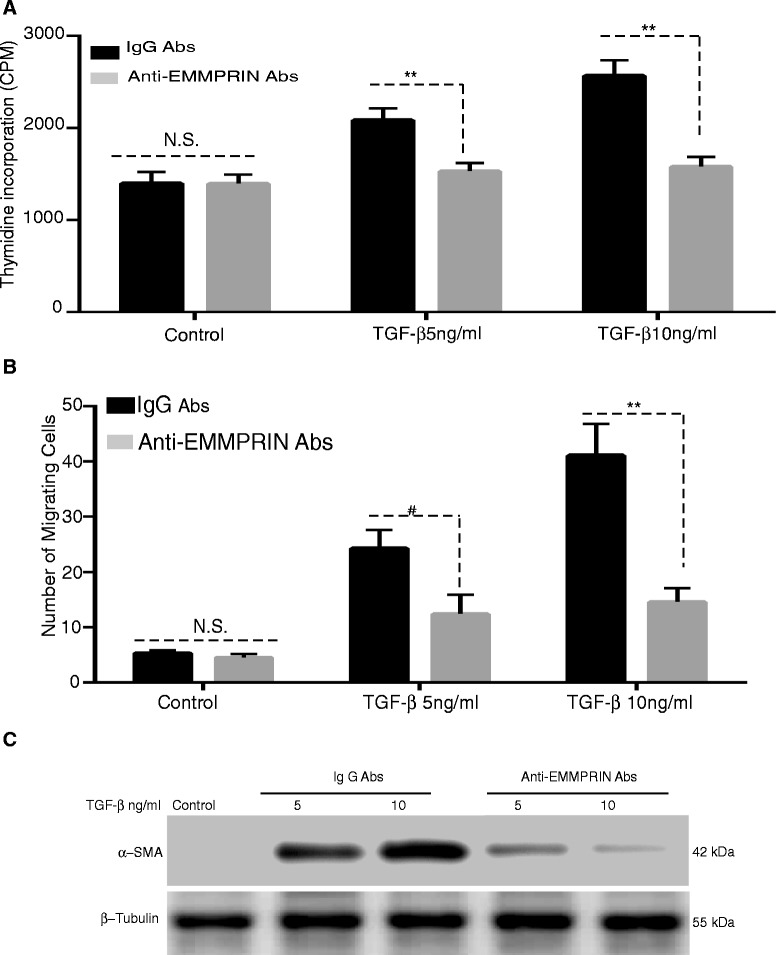
EMMPRIN blocking antibody attenuates TGF-β1 induced lung fibroblast proliferation, migration and differentiation to myofibroblast: NHLF were stimulated with TGF-β1 (0-10 ng/ml) in the presence of either EMMPRIN functional blocking antibody or IgG control antibody examined for (**a**) Cells proliferation using [3H] thymidine incorporation; (**b**) Cell migration using a modified Boyden chamber assay. Each bar represents means ± SEM of 3 independent experiments, each run in duplicate, # p = 0.002 or ** p < 0.001 comparing EMMPRIN functional blocking antibody to IgG control antibody treated cells; and (**c**) Cell differentiation using Western blot for α-SMA of cell lysates from NHLF treated with TGF-β1 (5 and 10 ng/ml) in the presence of either EMMPRIN functional blocking antibody or IgG antibody. β-Tubulin was used as the loading control. Image is a representative of 3 independent experiments, each run in duplicate

As shown in Fig. 6a, TGF-β1 significantly induced an increase in NHLF proliferation as evidenced by increased thymidine incorporation (p < 0.05). The EMMPRIN blocking antibody significantly inhibited TGF- β1 induced fibroblast proliferation. Using a transwell migration assay, TGF-β1 significantly induced an increase in NHLF cell migration and EMMPRIN functional blocking antibody significantly inhibited TGF- β1 induced NHLF migration (Fig. 6b). TGF-β 1 significantly induced an increase in NHLF differentiation to myofibroblasts as evidenced by an increase in α-smooth muscle actin. Similar to proliferation and migration, EMMPRIN blocking antibody significantly inhibited TGF- β1 induced fibroblast differentiation to myofibroblasts (Fig. 6c). These results imply that the effects of TGF- β1 on NHLF behavior can be attributed to an increase in EMMPRIN expression.

### Overexpression of EMMPRIN in NHLF results in activation of a Wnt/β-catenin signaling pathway

**Fig. 7:**
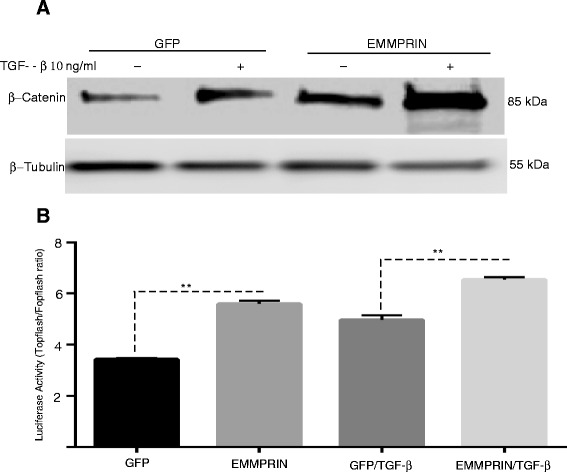
EMMPRIN Overexpression Increases Wnt/β-catenin signaling: (**a**) Representative Western blot for phosphorylated β-catenin of cells lysates from NHLF overexpressing either EMMPRIN/GFP or GFP treated with and without TGF-β1 (10 ng/ml). β-Tubulin was used as the loading control. Image is representative of 3 independent experiments, each run in duplicate. **b** Transient transfection of TOPFLASH, FOPFLASH, and control renilla luciferase reporter constructs into NHLF overexpressing either EMMPRIN/GFP or GFP then treated with TGF-β1 10 ng/ml. Each bar represents means ± SEM of 3 independent experiments, each run in duplicate, ** p < 0.001comparing GFP to EMMPRIN/GFP transfected cells

WNT/ β -catenin signaling pathway has been shown to play a major role in the development of experimental and human pulmonary fibrosis [27]. To explore the mechanism by which EMMPRIN enhances NHLF proliferation, migration, and differentiation, we examined the active status of β-catenin, which is an important factor in the canonical Wnt signaling pathway that can be induced by TGF- β1. Immunoblotting studies demonstrated an increased expression of phosphorylated β-catenin in cell lysate from EMMPRIN overexpressing NHLF compared to GFP transfected control cells. TGF-β1 treatment of EMMPRIN overexpressing NHLF further increased the expression of β- catenin (Fig. 7a). To validate the functional significance of increased β-catenin expression, Wnt signaling activity was measured by luciferase expression controlled by TOPFLASH. EMMPRIN overexpression in NHLF was found to induce an increase in TOPFLASH luciferase reporter activity when compared with GFP control-transfected cells (Fig. 7b). TGF-β1 treatment of EMMPRIN overexpressing cells induced an increase in TOPFLASH luciferase reporter activity when compared with GFP control cells (Fig. 7b).

## Discussion

EMMPRIN, which is usually expressed on tumor cells of epithelial origin, is responsible not only for induction of MMPs in neighboring stromal cells but also for transformation of quiescent fibroblasts to cancer-associated fibroblasts through direct tumor–stromal interactions [25]. Apart from its role on cancer cells, EMMPRIN expression is increased not only in lung fibrosis [23], but also in other types of tissue fibrosis including renal fibrosis [31], hepatic fibrosis [32] and corneal wound healing response [33]. EMMPRIN expression in all these situations is mainly localized in actively differentiating basal epithelial layer implying a role of EMMPRIN in epithelial stromal interactions [22]. The precise regulatory mechanism of EMMPRIN in lung fibrosis has not been elucidated. In this study, we examined EMMPRIN’s role in the regulation of pro-fibrotic phenotypic changes in human lung fibroblast by studying fibroblasts proliferation, migration, differentiation, and resistance to apoptosis. We present evidence that EMMPRIN overexpression significantly increases lung fibroblast proliferation, migration and differentiation to myofibroblasts. These findings were associated with activation of the Wnt/β-catenin signaling pathway. Additionally, we present evidence that TGF-β1 induces EMMPRIN expression in human lung fibroblasts. Finally, we demonstrated that inhibition of EMMPRIN using an EMMPRIN blocking antibody enhanced the apoptotic susceptibility of lung fibroblasts. Further, blocking EMMPRIN functionally inhibited lung fibroblast proliferation, migration, and differentiation to myofibroblasts in response to TGF-β1. Collectively, these findings indicate that increased EMMPRIN expression in the lung of IPF patients represents one mechanism by which lung fibroblasts acquire a proliferative, migratory and anti-apoptotic phenotype.

The data presented here suggests that TGF- β1 increases lung fibroblast proliferation, migration, and differentiation into myofibroblasts via upregulation of EMMPRIN expression, which may be another potential mechanism to explain the increased number of lung fibroblasts in pulmonary fibrosis. Our results are in agreement with other recent studies [22, 24] demonstrating that TGF-β1 contributes to the upregulation of EMMPRIN in normal epithelial cells and fibroblasts. Moreover, EMMPRIN, by inducing MMPs releases activate TGF- β1, which further upregulates EMMPRIN and creates a positive feedback regulation that potentially amplifies EMMPRIN’s effects [24, 34]. MMPs, specifically MMP-2, -9 and MMP-14, have been reported to activate TGF- β1 [14–18]. MMP-2 deficiency suppressed the activation of latent TGF-β and the Smad2/3 pathway in vivo and in vitro [17].

We also provide evidence that EMMPRIN overexpression induces resistance of NHLF to the apoptotic effects of Staurosporine, a known inducer of apoptosis. The EMMPRIN blocking antibody induced apoptosis of NHLF treated with TGF- β1. These data suggest that EMMPRIN acts as a fibroblast survival factor and TGF- β1 increases survival of lung fibroblasts via upregulation of EMMPRIN expression in these cells. These findings are supported by the report of Ma et al. [35] demonstrating that cell–cell contact mediated by EMMPRIN conferred apoptosis resistance in an E-cadherin dependent manner and down regulation of EMMPRIN inhibited cell–cell contact formation, which induced cell apoptosis. Xie et al also demonstrated that EMMPRIN overexpression protects human umbilical vein endothelial cells (HUVECs) from apoptosis [36]. Other studies have shown that EMMPRIN inhibits apoptosis by activating the FAK-PI3K-calcium (Ca^2+^) signaling pathway by interacting with α3β1 integrin and disrupts the nitric oxide /cGMP-mediated negative regulation of store-operated calcium entry, thus increasing the intracellular level of calcium [37]. The disruption of intracellular calcium homeostasis can disturb endoplasmic reticulum (ER) function and induce ER stress (ERS) [38].

We report in this study that EMMPRIN overexpression in NHLF induces fibroblast migration and EMMPRIN blocking antibody inhibits NHLF migration in response to TGF- β1. These results are attributed to the role of EMMPRIN in inducing MMPs and its interaction with a number of binding partners on the cell surface, including the hyaluronan receptor CD44 [39–43]. These interactions are critical for regulating cell migration, survival and proliferation, and events required in different processes such as wound healing [44]. A study by Toole and Slomiany [45] demonstrated that EMMPRIN overexpression plays an important role in cell migration and invasion via inducing hyaluronan (HA) production [45]. Overexpression of HA synthase 2 (HAS2) by lung fibroblasts produced an aggressive invasive phenotype leading to severe lung fibrosis and death after bleomycin-induced injury. Fibroblasts isolated from patients with IPF exhibited an invasive phenotype that was also dependent on HAS2 and CD44. EMMPRIN interaction with CD44 has been reported to potentiate cell survival pathways and the invasive phenotype of epithelial cells. EMMPRIN also promotes assembly of signaling complexes containing EMMPRIN, CD44, and EGFR in lipid raft like domains forming a positive feedback loop that may amplify invasiveness of epithelial cells [46].

Moreover, we provide evidence that EMMPRIN overexpression in NHLF promotes the differentiation of fibroblasts into myofibroblasts by inducing α-SMA expression and EMMPRIN blocking antibody inhibits TGF- β1 induced differentiation of fibroblasts to myofibroblasts. These data are supported by a previously reported study showing that EMMPRIN induced corneal fibroblast differentiation into myofibroblasts by inducing α-SMA through MMP-independent mechanisms [22]. Blocking EMMPRIN expression by small interfering RNA inhibited α-SMA and collagen gel contraction induced not only by EMMPRIN but also by TGF-β1, a major mediator of myofibroblast differentiation [22]. EMMPRIN and α- SMA were co-localized to the same cells in the stroma of pathological tissue, thus supporting a role for EMMPRIN in the differentiation of myofibroblasts in vivo. These data suggest that in addition to regulating the degradative potential of the myofibroblasts, EMMPRIN can also influence the contractile phenotype of these cells in an MMP independent manner and expand on the mechanism by which EMMPRIN remodels ECM during wound healing and tissue fibrosis.

Finally, we demonstrate that EMMPRIN overexpression in NHLF increased Wnt/β –catenin signaling, as shown by increased β -catenin expression in EMMPRIN overexpressing NHLF and increased TOPFLASH luciferase reporter activity. The Wnt/β catenin pathway is thought to play a major role in pulmonary fibrosis. Abnormal activation of Wnt/ β catenin pathway has been demonstrated in lung tissue of patients with IPF [47] and in an experimental model of bleomycin induced pulmonary fibrosis [48]. MMPs especially MMP-2, -7, -9, -14 and TGF – β1 have been known as Wnt/ β- catenin target genes [49–51]. Supporting our data is a study by Sidhu et al [26] demonstrating that EMMPRIN has a regulatory role over the Wnt/β -catenin signaling pathway. Increasing EMMPRIN expression upregulated the β -catenin signaling pathway and silencing EMMPRIN inhibited β -catenin signaling, cell migration, proliferation, anchorage-independent growth and tumor growth in a mouse tumor xenograft model [26].

## Conclusion

The present study demonstrates that TGF-β1 induces the release of EMMPRIN that in turn activates the β-catenin/canonical Wnt signaling pathway. EMMPRIN over expression in human lung fibroblasts contributes to the proliferative, migratory, and anti-apoptotic phenotype of lung fibroblasts that may contribute to the persistent fibro-proliferative state seen IPF. Further studies are needed to explain the mechanism by which EMMPRIN mediates the phenotypic changes in human lung fibroblasts and regulates TGF- β1 signaling.
